# Efficacy and safety of Vinflunine for advanced or metastatic urothelial carcinoma in routine practice based on the French multi-centre CURVE study

**DOI:** 10.1186/s12885-016-2262-9

**Published:** 2016-03-14

**Authors:** Jacques Médioni, Mario Di Palma, Aline Guillot, Dominique Spaeth, Christine Théodore

**Affiliations:** Medical Oncology Department, Hôpital Européen Georges Pompidou, 20, rue Leblanc, 75015 Paris, France; Department of Medicine, Gustave Roussy Institute, 114 Rue Édouard Vaillant, 94805 Villejuif, France; Lucien Neuwirth Institute of Cancerology, 108 B Avenue Albert Raimond, 42270 Saint-Priest-en-Jarez, France; Gentilly Oncology Centre, 2 rue Marie Marvingt, 54100 Nancy, France; Department of Oncology, Hôpital Foch, 92151 Suresnes, France

**Keywords:** Vinflunine, Metastatic, Urothelial carcinoma, Chemotherapy, Routine, Platinum pre-treated, CURVE (urothelial carcinoma patients treated by VinfluninE study-in French)

## Abstract

**Background:**

To retrospectively assess the efficacy and safety of Vinflunine (VFL) under routine conditions and identify overall survival (OS) prognostic factors.

**Methods:**

Twenty centres participated in the retrospective study (minimum 4 patients undergoing VFL treatment for advanced/metastatic UC after platinum-based regimen progression. Primary endpoint was OS. Secondary endpoints: progression-free survival (PFS), radiological response rate (RR) RECIST criteria and toxicity (CTC NCI v3).

**Results:**

These centres enrolled 134 patients. Prior chemotherapy (CT) lines (≥1 palliative): 1 and ≥2 in 69 % and 26 % of patients, respectively. Performance status (PS): 0, 1, 2 in 25 %, 46 % and 23 % of patients. Median OS = 8.2 months [6.5–9.4], PFS = 4.2 months and RR 22 %, median number of 5 cycles. In risk groups based on 0–3 presence of adverse prognostic factors (PS ≥1, haemoglobin ≤10 g/dl and liver metastasis), median OS: 13.2, 9.9, 3.6, and 2.4 months (*P* < .0001), respectively; 3.3 months (1.9–5.6) in PS ≥ 2 subgroup.

**Conclusion:**

This study reflects routine UC management and confirmed VFL patient efficacy. The drug is safe with gastro-intestinal and haematological prophylaxis. Analysis of prognostic factors for OS is consistent with pivotal trials.

## Background

Bladder cancer is a major health problem with an estimated 429,793 new cases and 165,068 related deaths in 2012, worldwide [1]. Transitional cell carcinoma of the urothelium (TCCU) is of particular concern in Western countries, with the highest incidence rates in Europe and North America. In 2012, bladder cancer was the fifth most frequent malignancy with 4,772 deaths in France [2].

Recurrence of non–muscle-invasive urothelial carcinoma of the bladder is common despite treatment; low and high grade tumours have recurrence rates of 50 %–70 % and more than 80 %, respectively. Stage progression occurs in 15 %–20 % of non–muscle-invasive cases [3]. It is a muscle-invasive disease with poor progression and survival. Patients have a significantly high risk for progression to regional and systemic disease. Regardless of treatment, the 5-year overall survival (OS) rate for patients is approximately 50 % [4]. For patients with metastatic disease, a cisplatin-based combination is the standard first-line treatment. However, approximately one patient out of two is not fit enough to receive cisplatin therapy and the alternative is usually a carboplatin-based regimen or a single agent. Median survival is approximately 14 months in cisplatin-eligible patients and less than 10 months for unfit patients. Although initial response rates are high in these patients, disease progression is common, creating a number of patients in need of effective second-line chemotherapy (CT) [5–7]. Until recently no standard of care was available for patients who failed a first systemic chemotherapy regimen. Studies exploring potential clinical activity of anticancer agents are mainly limited to non-randomized phase II trials without scientific evidence assessing a clinical benefit over supportive care [6, 7].

Vinflunine (VFL), a novel microtubule inhibiting agent, has been shown to be effective against a variety of solid tumour types including advanced TCCU. In vivo, VFL has shown to have a greater antitumor activity than other vinca alkaloids [8, 9]. VFL is the first and only agent that has to date been assessed in a randomised phase III trial in a second-line setting, compared with best supportive care (BSC). In fact, prior to the development of this agent, there was neither an approved agent nor any established standard second-line therapy available in this setting. VFL plus BSC achieved a 2.6 month overall survival (OS) advantage in patients with advanced TCCU over BSC alone [10].

In Europe, VFL has been approved as a treatment option for patients with advanced urothelial cancer who failed a prior platinum-containing regimen [10–13]. VFL was introduced in France in September 2010 and has been integrated into the French and European guidelines [6, 7, 14–16].

The first aim of the CURVE (“Carcinoma of the Urothelium: patients treated by VinfluninE”) study was to retrospectively assess the efficacy and safety of VFL in second-line treatment for advanced or metastatic urothelial carcinoma in daily routine practice. The secondary objective was to confirm whether the prognostic factors for OS identified across the pivotal trials (performance status (PS), haemoglobin level and liver metastasis) were relevant or not in routine practice as well as to evaluate other possible prognostic factors for OS [17].

## Methods

### Data source

Eighty-eight French centres (i.e. private clinics, university hospitals, private hospitals) that had treated at least four patients with VFL for advanced or metastatic cancer after failure of platinum-based (CT) during 2011 (January-December) were contacted and 22 centres agreed to participate in the study. Prior written consent was not required from patients according to French regulations as this was an anonymous non-interventional study. Nevertheless, patients received complete information regarding the series and the anonymous collection of data for research. If a patient refused participation, the registration was not performed. Data were collected between December 2011 and March 2012.

### Ethics committee approval

French Government approval, in compliance with the Helsinki Declaration, was received to use the patient data for the French multi-centre CURVE study from: 1- The Advisory Committee for the Treatment of Information – Ministry of Higher Education gave their approval on the 17^th^ September, 2012, Dossier N° 12.526. 2- The National Commission of Informatics and Civil Liberty gave their approval on the 19^th^ October, 2012 Decision DR-2012-504, authorization demand N° 912480.

Characteristics of patients at the beginning of treatment therapy with VFL included age, performance status (PS), metastatic sites (i.e. liver, bone, lung, skin and brain), renal/liver function, relevant medical history i.e. gastro-intestinal chronic disease, cardiac disease, or previous treatment. The disease history was recorded, as well as, the number of previous treatment lines, regimen and duration of prior CT. VFL treatment modalities (starting dose, dose-escalation or reduction, number of cycles), the haematological and non-haematological adverse events were also recorded [18] including reasons for stopping treatment. Efficacy parameters i.e. response rate (radiologic assessment considering RECIST 1.1), progression-free survival and overall survival were included in the database.

### Patient inclusion criteria

Only patients 18 or over, who had previously received a single-agent therapy with VFL for advanced or metastatic TCCU after progression to a platinum-based CT were enrolled in the study. Treatment with VFL was administered from 01/01/11 to 31/12/11.

### Assessment criteria efficacy

Primary endpoint was OS, i.e. time between treatment start and date of last follow-up or death. Progression-free survival was defined as the time between treatment start and disease progression (defined as the appearance of two or more lesions on a bone scan, or progression of visceral metastases by computer tomography scan according to RECIST 1.1.) or death. Efficacy was also measured using radiological response rate (complete response [CR] + partial [PR] response, and disease control (CR + PR + Stable Disease [SD]) were based on the RECIST 1.1 criteria.

### Tolerance assessment

The following adverse events were routinely recorded: neutropenia, febrile neutropenia, thrombocytopenia, anaemia, constipation, nausea/vomiting, infection, fever, asthenia/fatigue, neuropathy, stomatitis, mucositis, and abdominal pain. Other adverse events, based on a standard criteria were also recorded [18].

### Prognostic factors

Several potential pre-treatment prognostic factors for OS were investigated in univariate/ multivariate analyses: PS (0 or ≥1), previous history of pelvic radiotherapy, haemoglobin level (≤10 or >10 g/dl), creatinine clearance (≤60 or >60 ml/min), liver function (abnormal or normal), previous lines of CT (≤1 or >1), metastases at diagnosis, visceral metastases, liver metastases, lung metastases, previous treatment by cisplatin, previous treatment by cisplatin versus carboplatin, progression free interval before VFL (<6 or ≥6 months), previous surgery (bladder or kidney/ureter).

### Statistical analysis and quality control

Data were analysed (SAS system software version 9.3). Descriptive methods were used to present the data: quantitative variables were described either by mean plus standard deviation, or median with the range of values; qualitative variables were expressed by frequency and percentages, confidence interval of 95 %.

Survival data univariate analysis was performed using a log-rank test and multivariate analysis with a Cox proportional hazard model. Hazard ratios were estimated using the Cox proportional hazard model.

A scientific committee of the five authors controlled protocol conditions and reviewed all data prior to statistical analysis.

## Results

### Data source

Among the eighty-eight centres contacted twenty (i.e. 50 % university hospital or cancer centres, 35 % private clinics and 15 % private hospitals) agreed to participate in the study with data collected from 134 patients.

### Previous medical history of patients

In the intention to treat (ITT) population the sex ratio (female/male) was 11.2/88.8, median age 65.3 [range 42.1-88.2] with patients ≥65 years and ≥ 75 years old representing 52 % and 21 %, respectively. PS was 0 in 34 patients (25 %), 1 in 62 (46 %) and PS ≥ 2 in 31 (23 %); information was missing for 7 patients. (Main Patients Characteristics see: Table 1.)

**Table 1:** Patients characteristics and potential prognostic factors

ITT population	*N* = 134
Gender (%) F/ M	15 (11.2)/119 (88.8)
Median Age (years, min/max)	65.3 [42.1–88.2]
[35–50 [N (%)	7 (5.2)
[50–65 [N (%)	57 (42.5)
[65–75 [N (%)	42 (31.3)
≥75 N (%)	28 (20.8)
Performance Status N (%)	
0/1/ ≥ 2/Na	34 (25.4)/62 (46.3)/31 (23.1)/7 (5.2)
Metastases N (%)	
Lung/Liver/Bone/Lung + Liver/Other	52 (38.8)/38 (28.4)/32 (23.9)/76 (56.7)/20 (14.9)
Creatinine Clearance N (%)	
>60 ml/mn/[40–60] ml/mn/ < 40 ml/mn/Na	67 (50.0)/51 (38.1)/13 (9.7)/3 (2.2)
Haemoglobin N (%)	
<= 10 (g/dl)/ > 10 (g/dl)/Na	32 (23.9)/100 (74.6)/2 (1.5)
Liver Function N (%)	
Normal/Abnormal/Na	114 (85.1)/13 (9.7)/2 (1.5)
Chronic Constipation N (%)	
No/Yes/Na	127 (94.8)/6 (4.5)/1 (0.7)
Prior Radiotherapy N (%)	28 (20.9)
Progression within 6 months after prior CT N (%)	112 (83.6)
Number of Advanced or Metastatic CT at Baseline [N, (%)] Total pts	128 (95.5)
1 CT lines	93 (69.4)
2 CT lines	31 (23.1)
3 CT lines	4 (3)
Patients with perioperative CT alone at Baseline [N, (%)]	5 (3.7 %)

All patients received prior chemotherapy, with the majority (95.5 %) for advanced or metastatic disease. Twenty-six percent (*n* = 35) of patients received ≥ 2 prior chemotherapy lines for an advanced/metastatic urological cancer and 6 patients (4.5 %) received only one prior chemotherapy regimen as perioperative treatment in the neoadjuvant (1 patient) or adjuvant setting (5 patients).

All but one patient received a prior platinum salt: a cisplatin-based regimen was administered in 69.4 % of patients and 50.7 % received a carboplatin-based chemotherapy, considering that some patients received several prior CT lines (Table 1).

Progression within 3 months and 6 months after prior CT was observed in 54 % and 82 % of patients, respectively. Prior local therapies in the ITT population were: 28 patients (20.9 %) had undergone prior radiotherapy (RT), 19 for bladder cancer; 112 patients (83.6 %) had undergone prior surgery, mainly localized to the bladder alone (76 patients), or 22.4 % for the upper urinary tract.

### Vinflunine treatment modalities

The median number of cycles was 5 [1; 23] with a median duration of treatment by VFL of 3.1 months [0.03; 15.2]. Patients started i.v. VFL at either 320, 280, 250 or < 250 mg/m^2^ dose for a 20 min infusion, 3 weekly-based. VFL starting dose was ≥ 280 mg/m^2^ in 81 % (*n* = 108) of patients with a median initial dose of 280 mg/m^2^ (min: 120 mg/m^2^; max: 320 mg/m^2^). In fact, 54.5 % of patients initially received 280 mg/m^2^ and 26.1 % received 320 mg/m^2^. The relative dose intensity reached the median value of 92.7 % (18.8–125.7). At least one dose reduction occurred in 16 % of patients.

In terms of treatment duration, 17.9 % of patients had a completion of cycles initially scheduled. Treatment was stopped for toxicity in only 6 % of patients but primarily stopped for disease progression or death (63.5 %).

### Tolerance and safety

In patients, most frequent grade III-IV toxicities (whatever the drug relationship) were asthenia and fatigue (21 %, *n* = 28), neutropenia (17 %, *n* = 23), anaemia (8 %, *n* = 11), constipation (8 %, *n* = 11) and abdominal pain (5 %, *n* = 7). Other grade III and IV toxicities were observed with a frequency below 5 % of patients. Occlusion occurred in one patient with no toxic deaths. Prophylaxis against vomiting and constipation were prescribed in 92 % and 86 % of patients respectively. Table 2 shows the reported adverse events (all grades and grade III-IV).

**Table 2:** Most common adverse events (regardless of the drug-relationship)

Adverse event	All grades N (%)	Grades 3/4 N (%)
	Haematology	
Anaemia	60 (44.8)	11 (8.2)
Leucopenia	23 (17.2)	7 (5.2)
Neutropenia	34 (25.4)	23 (17.2)
Thrombocytopenia	14 (10.4)	4 (3)
Febrile Neutropenia	5 (3.7)	4 (3)
	NON HAEMATOLOGY	
Asthenia, Fatigue	74 (55.2)	28 (20.9)
Constipation	55 (41.0)	11 (8.2)
Abdominal pain	21 (15.7)	7 (3)
Subocclusive syndrome	5 (3.7)	3 (2.1)
Neuropathy	10 (7.5)	1 (0.7)
Toxic death	0	0

### Primary end point - OS

**Fig. 1:**
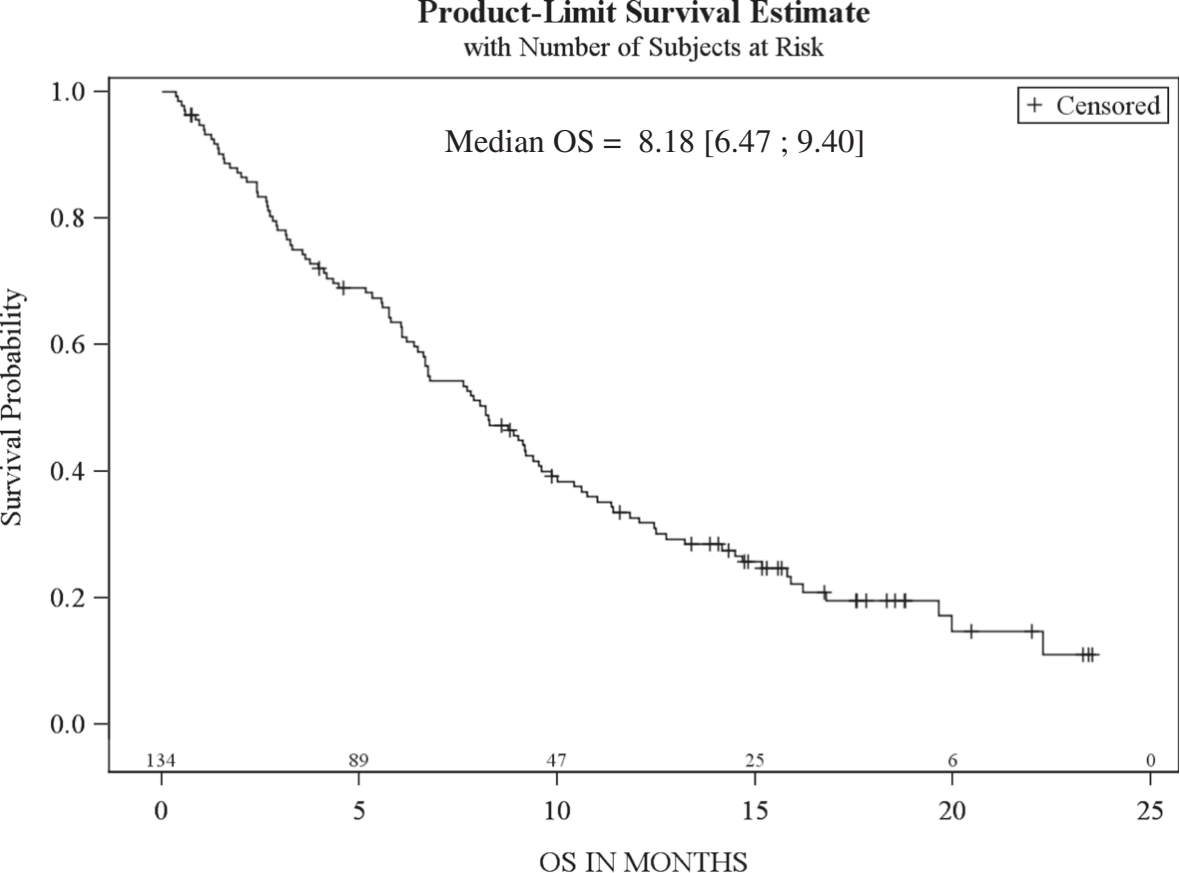
Overall Survival (ITT, *n* = 134)

After a median follow-up of 17.6 months [95 % CI 15.3 – 18.8] the median OS for the entire population was 8.2 months [95 % CI 6.5 - 9.4] (Fig. 1). Among the 31 patients with PS ≥ 2 at treatment initiation, median OS was 3.3 [95 % CI 1.9-5.6].

Following VFL, 62 patients (46 %) received further treatment, from 1 (46 patients) to 3 additional lines, mainly CT (59 patients).

### Secondary efficacy criteria

**Fig. 2:**
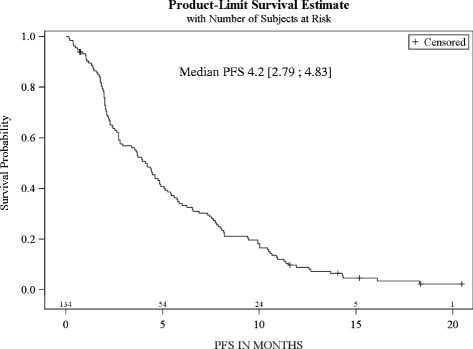
Progression-free Survival (ITT, *n* = 134)

Median PFS was 4.2 months [2.8–4.8] (Fig. 2). The RR was 22 % with 5 % and 17 % of complete and partial responses, respectively (Table 3). The disease control rate was 51 % with a median duration of 7.7 months [6.0–9.4].

**Table 3:** Efficacy results

ITT population	*N* = 134
Best Overall Response	
Complete Response (n, %)	7 (5.2 %)
Partial Response (n, %)	23 (17.2 %)
Stable Disease (n, %)	38 (28.4 %)
Response Rate (CR + PR) (%, range)	30 (22.4 %)
Disease Control (CR + PR + SD) (%, range)	68 (50.7 %)
Median duration of response (month, range)	4.9 [3.2–6.5]
Median duration of stabilization (month, range)	7.8 [5.7–9.4]

#### Independent prognostic factors for OS

Based on univariate analysis at baseline, four factors were significantly associated with OS: PS (0 versus ≥1), baseline haemoglobin level (>10 versus ≤10 g/dl), liver function (normal versus abnormal) and liver metastasis (yes versus no). For PS values 0 or ≥1, OS was 14.5/ 6.1, *p* = 0.0002 hazard ratio (HR) 0.40; for baseline haemoglobin >10 g/dL or ≤ 10 g/dL, OS 9.6/2.4 months, *p* < .0001, HR 0.30; presence of baseline liver metastasis (yes or no), OS was 9.4/5.6 months, *p* = 0.0059 (HR) 0.55; and according to the normality or abnormality of liver function, OS was 8.7/1.6 months, *p* = 0.0001 (HR) 0.31.

All factors except liver function in the multivariate analysis had a statistically significant effect on OS and none of the other parameters were significantly correlated with OS.

### Distribution of patients according to risk groups

**Fig. 3:**
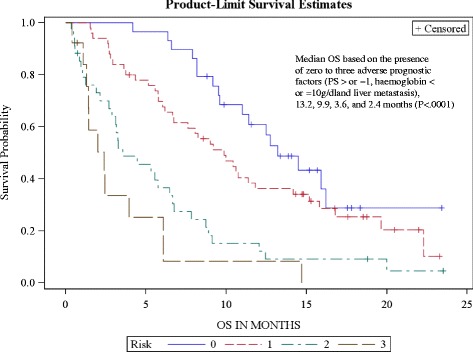
Overall survival according to risk group (*n* = 126)

According to previously published models, median OS was assessed based on the presence of zero, one, two or three risk factors (PS ≥1, haemoglobin ≤10 g/dl and liver metastasis); these results are shown in Table 4 with 21.6 % of the 134 patients in risk group 0, 37.3 % in risk group 1, 25.4 % in risk group 2, 9.7 % in risk group 3 and for 6 % of patients information not available (Fig. 3)

**Table 4:** Distribution of patients according to risk groups

*P* < 0.0001 HR 1.98	*N* = 134	OS Median months [95 % CI]
Risk 0	29	13.2 [9.6 – NR]
Risk 1	50	9.9 [6.7 – 11.8]
Risk 2	34	3.5 [2.7– 6.5]
Risk 3	13	2.4 [1.1 – 6.17]
Missing^a^	8	

Patients group Risk 0, 1, 2 or 3: patients having 0, 1, 2 or 3 adverse factor respectively

Prognostic factors: PS ≥ 1, haemoglobin ≤10 g/dl, presence of liver metastasis at baseline

^a^ Patients with at least one missing prognostic factor

## Discussion

To date, the CURVE study represents the largest cohort of patients following cytotoxic treatment after failure of a prior platinum-based CT regimen in urothelial carcinoma.

The aims were not only to report on patients treated with vinflunine in routine daily practice, but also conditions of vinflunine administration as well as provide data on efficacy and toxicity. The analysis based on real life conditions of possible prognostic factors of OS is useful when compared to previously published data from clinical trials [17].

This survey reflects the current second-line management of metastatic UC in France with some patients exhibiting worse conditions (age and PS) that are often reported in current practice or clinical trials. In fact, the median age was 64.2 years in the pivotal phase III study, and only patients with PS 0 and 1 were enrolled the phase II and III trials in contrast to 23 % of PS 2 reported in our study [10–12].

The obvious limitation of this study is that it was retrospective. Moreover, not all the data concerning the patients treated in France with vinflunine during the year 2011 are reported in this study. Nevertheless, the protocol followed a strictly predefined methodology under the supervision of the scientific committee, all centres with a major recruitment of bladder cancer were contacted, and all those that participated had treated at least 4 patients, which reflects their experience in treating VFL patients. The series were considered to be exhaustive in each participating centre and no discrepancy was observed when the pre-study estimated number of cases was compared to the registered patients. No patient selection bias for VFL related outcomes was detected during the quality control assessment.

Regarding treatment modalities in daily practice, 81 % of patients were treated with VFL starting doses of 280 or 320 mg/m^2^ as per SmPC as recommended by the official guidelines. There was in fact a high median number of cycles, i.e. 5 cycles as compared to 3 in the phase III study. One patient received up to 25 cycles. This information on treatment duration is reassuring regarding VFL tolerance under routine conditions.

Toxicity was manageable: i.e. main grade 3 or 4 toxicities were neutropenia 17 %, anaemia 8 %, asthenia/fatigue 21 % and constipation 8 % with no toxic deaths.

Two similar European studies reporting VFL efficacy and safety in routine practice were recently reported. In a prospective non-interventional study, 77 unselected patients (within the VFL market authorization conditions of PS 0–1) with advanced or metastatic urothelial carcinoma were treated in Germany [19]. The mean number of administered cycles of VFL was 4.7 and 34 % of patients received the 6 planned cycles. Most frequent grade 3 or higher haematological toxicities were leucopenia (17 %) and anaemia (6.5 %). As regards prophylaxis against nausea/vomiting or against constipation, grade 3 or higher non-haematological toxicities were constipation (5 %) and nausea/vomiting (5 %). Median OS time was 7.7 months and disease control rate was 53 %. These prospective results are similar to those in the retrospective CURVE study.

Results from another ongoing Spanish study were also presented in 2013. Chirivella et al. reported their initial results based on 66 patients with only 5 PS 2. Median duration of treatment was also 5 cycles [1, 17, 20] with some long lasting treatments. Grade 3 or higher toxicities were: 9 % neutropenia, 6 % constipation and abdominal pain, 6 % nausea or vomiting with an OS of 10.6 months. These toxicity and efficacy results are quite similar to the CURVE study. The Spanish population was mostly elderly (median age 67 years) but with better performance status (only 7 % PS2). The difference in PS could possibly explain the more favourable OS in the Spanish cohort while other efficacy outcomes and toxicity were similar.

In the phase III controlled randomised clinical trial reported by Bellmunt et al. in 2009 updated in 2013, after a median follow-up of 45.4 months, median OS was 6.9 m and 4.3 m for VFL plus BSC versus BSC alone, respectively in the modified ITT population. [21]. The median age was lower than in the CURVE study for the VFL treated group (64.2 years) and PS was restricted to PS 0 or 1. Similarly to the CURVE study, progression or relapse within 6 months following prior CT accounted for 83 % of patient cases and liver metastasis were present in 29 %. In contrast, a baseline haemoglobin below 10 g/dL was reported at a higher rate (86 % of patients) in the phase III study which probably represents, apart from an initial more restricted patient selection (including PS and only one prior chemotherapy line), the main difference between the populations’ characteristics.

In the Bellmunt et al. 2009 study, the main grade 3 or 4 toxicities for VFL + BSC were neutropenia (50 %), anaemia (19 %), fatigue (19 %), constipation (16 %), and febrile neutropenia (6 %) [10].

The toxicity was less pronounced in our daily experience than in the pivotal trial. This was a reproducible observation across the other studies performed in routine practice, either prospective or retrospective, and could be linked to the high rate of constipation prevention with laxatives and dietary measures at each cycle of VFL administration. In our study, 63 % of patients received a prophylaxis with G-CSFs thus preventing neutropenia.

The current analysis of prognostic factors for OS is consistent with the data published from the pivotal study. Bellmunt et al. showed that PS, haemoglobin, liver metastasis, were prognostic factors of overall survival, but also liver enzymes AST and alkaline phosphatases had a lesser relationship to OS. In the same phase III clinical trial, based on multivariate analysis, the internal validation identified PS of more than 0, a haemoglobin level less than 10 g/dL, and the presence of liver metastasis as the main adverse prognostic factors for OS. External validation confirmed these prognostic factors. We also assessed OS in the four subgroups of the published model based on the presence of zero, one, two or three similar prognostic factors with the median OS times of 13.2, 9.9, 3.6, and 2.4 months (*P* < .0001), respectively. The current analysis is consistent with the phase III findings where median OS was 14.2, 7.3, 3.8 and 1.7 months, respectively. This is roughly comparable even if the presence of PS 2 patients in the CURVE study may have played a role in the later risk groups [17].

Our study further confirms the value of PS, haemoglobin level and liver involvement as useful prognostic factors for OS. In fact, both the presence of liver metastasis and liver dysfunction were significantly correlated with OS in the univariate analysis. The effects of haemoglobin level and liver involvement was confirmed in the multivariate analysis although the presence of liver metastasis and liver function are not clinically independent parameters. The group of patients with 0 or 1 prognostic factors had the longest OS in our series and probably benefited the most by vinflunine treatment. Therefore, patients should be treated at an early stage after platin relapse, to avoid anaemia and alteration in performance status.

Yafi et al., in 2011, reported that no standard therapy had been established for patients who recur or are refractory to first-line therapy and that second-line VFL treatment, by way of superiority over best supportive care, has shown promise in a phase III trial [22]. The positive treatment effect of VFL on survival shown in the first publication of the phase III pivotal trial was recently confirmed in a recent up-dated report including a long term analysis. The results are consistent over time and confirm that VFL is a valuable option in patients with advanced TCCU after failure of platinum-based regimens. In addition, some long term survivors treated with vinflunine were observed for as long as 40 months follow-up (none in the BSC group). This is consistent with the long lasting treatments that were reported in routine practice [21].

Finally, several other clinical trials with VFL in bladder cancers are ongoing, notably regarding first line treatment for patients unfit for cisplatin-based chemotherapy. An international study (JASINT1) should provide data comparing vinflunine in association with gemcitabine as well as vinflunine with carboplatin [23]. In 2014 a study of 102 patients, by Castellano et al. in Spain, evaluated the safety and effectiveness of VFL in patents with TCCU after failure of one platinum-based systemic therapy in clinical practice with similar results to our study [24]. In their series they reported that 65.7 % of patients demonstrated a clinical benefit with VFL and an OS for all patients of 10 months (range 7.3–12.8). Recently, in agreement with our results, Retz et al. concluded: “A systematic gastrointestinal prophylaxis is strongly recommended to achieve a good safety profile. The vinflunine starting dose of 320 mg/m^2^ was most efficacious with a median OS of 10.4 months and should therefore be considered in all eligible patients [25]. This study adds further support to the EAU recommendation for the use of vinflunine as second-line therapy in advanced UCC after failure of platinum-based treatment”.

## Conclusion

The CURVE study was a major retrospective study in an unselected population of patients with pre-treated advanced or metastatic urothelial carcinoma. VFL efficacy as previously reported in the phase III pivotal study was confirmed or surpassed considering all criteria in real life patients. The drug was safe, as all the gastro-intestinal prophylactic recommendations were widely followed. Also the study was able to provide efficacy data for performance status 2 patients. Prognostic factors for OS in routine practice (performance status, haemoglobin, liver metastasis) were similar as those previously reported after the phase III trial. Risk group of patients with 2 or 3 adverse prognostic factors had a short median survival time but the presence of PS2 patients may have impacted the findings. In patients with or without one adverse prognostic factor, the median survival time ranged from 10 to 13 months.
